# Difficulty leading interpersonal coordination: towards an embodied signature of social anxiety disorder

**DOI:** 10.3389/fnbeh.2014.00029

**Published:** 2014-02-06

**Authors:** Manuel Varlet, Ludovic Marin, Delphine Capdevielle, Jonathan Del-Monte, R. C. Schmidt, Robin N. Salesse, Jean-Philippe Boulenger, Benoît G. Bardy, Stéphane Raffard

**Affiliations:** Movement to Health Laboratory, EuroMov, Montpellier 1 UniversityMontpellier, France; The MARCS Institute, University of Western SydneySydney, NSW, Australia; University Department of Adult Psychiatry, Hôpital de la Colombière, CHU Montpellier, Montpellier 1 UniversityMontpellier, France; INSERM U-888Montpellier, France; Epsylon, Laboratory Dynamic of Human Abilities & Health Behaviors, Department of Sport Sciences, Psychology and Medicine, University of Montpellier & St-EtienneFrance; Department of Psychology, College of the Holy CrossWorcester, MA, USA; Institut Universitaire de FranceParis, France

**Keywords:** social anxiety disorder, social coordination, interactional synchrony, leader, sensorimotor signature, motion capture

## Abstract

Defined by a persistent fear of embarrassment or negative evaluation while engaged in social interaction or public performance, social anxiety disorder (SAD) is one of the most common psychiatric syndromes. Previous research has made a considerable effort to better understand and assess this mental disorder. However, little attention has been paid to social motor behavior of patients with SAD despite its crucial importance in daily social interactions. Previous research has shown that the coordination of arm, head or postural movements of interacting people can reflect their mental states or feelings such as social connectedness and social motives, suggesting that interpersonal movement coordination may be impaired in patients suffering from SAD. The current study was specifically aimed at determining whether SAD affects the dynamics of social motor coordination. We compared the unintentional and intentional rhythmic coordination of a SAD group (19 patients paired with control participants) with the rhythmic coordination of a control group (19 control pairs) in an interpersonal pendulum coordination task. The results demonstrated that unintentional social motor coordination was preserved with SAD while intentional coordination was impaired. More specifically, intentional coordination became impaired when patients with SAD had to lead the coordination as indicated by poorer (i.e., more variable) coordination. These differences between intentional and unintentional coordination as well as between follower and leader roles reveal an impaired coordination dynamics that is specific to SAD, and thus, opens promising research directions to better understand, assess and treat this mental disorder.

## Introduction

Social anxiety disorder (SAD) is one of the most prevalent mental disorders, with estimates of its lifetime prevalence at about 7–13% (Furmark, 2002). SAD is defined by a persistent fear of embarrassment or negative evaluation while engaged in social interaction or public performance and tends to be followed by avoidant behavior (Lecrubier et al., 2000). SAD is associated with significant functional impairment in daily activities (Aderka et al., 2012a), social relationships (Wittchen et al., 2000), reduced quality of life (Safren et al., 1996), as well as increased risks of comorbid disorders such as depression, other anxiety disorders and alcohol abuse (Schneier et al., 1989; Merikangas and Angst, 1995; Stein et al., 2001). Accordingly, previous research made considerable efforts to better understand and assess this mental disorder.

However, very little attention has been paid to nonverbal behaviors of patients with SAD when interacting with other people. This is surprising given that several studies have demonstrated close relationships between the bodily behavior of interacting people and their mental states during social interactions (Chartrand and Bargh, 1999; Sebanz et al., 2006; Marsh et al., 2009; Wiltermuth and Heath, 2009). Indeed, the spatiotemporal organization of bodily movements (i.e., interpersonal or social motor coordination) can reflect feelings such as connectedness, social rapport or cohesion (Bernieri, 1988; Hove and Risen, 2009; Marsh et al., 2009; Miles et al., 2010). Interpersonal coordination can also reflect, for example, the social motives of the individuals (Lumsden et al., 2012). Individuals with a pro-social orientation spontaneously coordinate with others to a greater extent than those with a pro-self orientation. The coordination of arm, head or postural movements of people talking or acting together can thus directly reflect their mental states, suggesting that it could also reveal their potential SAD. Although there is no evidence yet of interpersonal motor coordination disorders in SAD, irregular gaze, posture or arm movements have been reported when patients are involved in social exchanges (Turner et al., 1986; Fydrich et al., 1998). Nevertheless, the movement coordination of SAD patients has only been examined at the individual level so far, independently of their coordination or synchronization with the movements of the others. Moreover, these behavioral abnormalities have been mainly quantified by using observational methods that are limited due to their qualitative and subjective nature. In particular, it is not possible for such methods to evaluate continuous and subtle changes over time of the spatiotemporal organization (i.e., coordination) of the movements of two or more people (Schmidt et al., 2012). In contrast, several studies with healthy people have used motion capture systems for objective and accurate measurement of social motor coordination, which has allowed a high-quality characterization and understanding of the underlying processes.

These studies demonstrated that social motor coordination between two or more people can be understood as constrained by the dynamical entrainment processes of coupled oscillators (see Schmidt and Richardson, 2008; Schmidt et al., 2011 for reviews). It has been demonstrated in a variety of coordination tasks that the movements of people are preferentially attracted toward in-phase and anti-phase patterns of rhythmic coordination, that is, characterized respectively by movements in the same and opposite directions and relative phase values of 0° and 180° (Schmidt and O’Brien, 1997; Richardson et al., 2007; Tognoli et al., 2007; van Ulzen et al., 2008; Varlet et al., 2011). Of particular note is that in-phase rhythmic coordination is more stable than anti-phase coordination, as indicated by lower levels of relative phase variability for in-phase compared to anti-phase coordination (Richardson et al., 2007). In unintentional coordination situations, as soon as visual information is exchanged between people such as when talking or walking together, their movements spontaneously and intermittently synchronize toward these two patterns of coordination (Schmidt and O’Brien, 1997; Richardson et al., 2007; van Ulzen et al., 2008). In intentional coordination situations, among all the possible patterns of coordination, only in-phase and anti-phase patterns can be stably maintained over time (Schmidt et al., 1990, 1998; Richardson et al., 2007). Moreover, previous research has shown that the emergence and stability of such unintended or intended coordination is closely linked to mental contents such as affiliation, rapport, social competences and social motives (Schmidt et al., 1994; Hove and Risen, 2009; Miles et al., 2010) as well as mental disorders such as schizophrenia and autism (Varlet et al., 2012b; Marsh et al., 2013).

Previous research has also shown that the stability of such coordination depends on how people visually pick up information about the movements of each other, which influences the strength of the coupling (informational linkage) underlying the coordination. It has been demonstrated that a stronger coupling, and thus more stable coordination, occurs when the movements of the other person are (a) perceived using central vision compared to peripheral vision (Richardson et al., 2007) and (b) visually tracked with the eyes (Schmidt et al., 2007; Varlet et al., 2012a). These results are of particular interest in view of the impairments of patients suffering from SAD. SAD results in abnormal gaze behaviors in social tasks, corresponding to both avoidant and vigilant patterns of attention varying over the time course of stimulus presentation (Mansell et al., 1999; Chen et al., 2002; Mogg and Bradley, 2002; Mühlberger et al., 2008; Schofield et al., 2012). Although these impairments have been mainly demonstrated for face perception, they suggest that SAD might result in weaker visual coupling strength, and hence, weaker social motor coordination.

Furthermore, previous research on social motor coordination has also demonstrated that the stability of the coordination depends on the difference between the preferred movement frequencies of participants (i.e., difference between their self-selected tempos) (Amazeen et al., 1995; Jeka and Kelso, 1995; Sternad et al., 1995; Fuchs et al., 1996). More stable coordination occurs when the preferred movement frequencies of participants, which highly depends on mechanical properties of the body segments involved, are similar. This effect has been successfully demonstrated using a hand-held pendulum paradigm because the preferred movement frequencies of participants can be manipulated by changing the inertial loading of the pendulums (Amazeen et al., 1995; Schmidt et al., 1998; Coey et al., 2011). Masses attached closer to the top or the bottom of the pendulums result in faster or slower (respectively) preferred movement frequencies. The stability of the coordination increases when participants swing similar pendulums and decreases when they swing different pendulums (Schmidt et al., 1998).

Interestingly, in intentional coordination tasks, the preferred movement frequencies of participants also influence how they lead or follow in the coordinated state (Schmidt et al., 1998; Richardson et al., 2007). The participant having the faster preferred frequency tends to lead the coordination whereas the participant with the slower preferred frequency tends to follow. Past research has also found that the degree of lagging or leading is amplified when coupling is weaker (Schmidt et al., 1998; Richardson et al., 2007). In view of this previous research, if SAD resulted in weaker visual coupling strength, the leader and follower positions would be exaggerated as indicated by larger phase shifts from the intended (i.e., in-phase or anti-phase) pattern of coordination. It is also possible, however, that potential social motor impairments in SAD may depend on the leader and follower role of the patient. The influence of SAD may reveal itself most when the patient has to lead the coordination. Supporting this prediction is previous research that has shown that patients suffering from SAD have poorer leadership skills (Bernstein et al., 2008). Moreover, additional research has shown that being in a leader position increases scrutiny and thus causes greater anxiety, which is known to degrade motor performance (Weinberg and Hunt, 1976; Lecrubier et al., 2000; Hatfield et al., 2013).

The goal of the current study was to investigate whether a disruption of social motor coordination occurs in SAD. We compared the unintentional and intentional coordination of patients diagnosed with SAD (DSM-IV criteria) with the coordination of control participants using a task that involved the swinging hand-held pendulums (Schmidt and O’Brien, 1997; Schmidt et al., 1998; Varlet et al., 2012b). In addition to this group comparison, we also examined the correlations between the stability of coordination performed by participants and their SAD severity as assessed by the Liebowitz Social Anxiety Scale (LSAS; Heimberg et al., 1999; Fresco et al., 2001), which quantifies fear and avoidance across a variety of social and public situations. A disturbance of the coordination was expected in SAD in view of the different abnormal social behaviors previously reported in this disorder, and more specifically, the impairments of visual social perception (Mansell et al., 1999; Chen et al., 2002; Mogg and Bradley, 2002; Mühlberger et al., 2008; Schofield et al., 2012). It is also possible that these disruptions would depend on the pendulum being swung by the patient, which constrains the preferred movement frequency performed, and hence, whether the patient was to lead or follow in the coordination condition.

## Materials and methods

### Participants

Nineteen patients diagnosed with SAD participated in the study. Diagnoses were made according to the Diagnostic and Statistical Manual of Mental Disorders, Fourth Edition (DSM-IV) criteria (American Psychiatric Association, 1994; First et al., 1997), using structured clinical interviews (SCID-IV). All social phobics were outpatients recruited from the University Department of Adult Psychiatry in Montpellier. Exclusion criteria for social phobia participants were: (a) known neurological disease; (b) developmental disability; (c) substance abuse in the past 3 months; or (d) current major depressive episode. The 19 patients were matched with 19 *matched control participants* for age, sex, education and premorbid IQ, as estimated by the French adaptation of the National Adult Reading Test (fNART; Mackinnon and Mulligan, 2005) (all *p*-*values* > 0.05) (see Table 1). During the experiment, the patients were randomly paired with *synchronization partners 1* to compose the SAD group and *matched control participants* were paired with *synchronization partners 2* to compose the control group. *Synchronization partners 1* and *synchronization partners 2* were matched for age, sex, education and premorbid IQ (all *p*-*values* > 0.05). Controls (*Matched control participants* and *synchronization partners 1 and 2*) were excluded if they had: (1) a history of social phobia or other anxiety disorder, bipolar or psychotic disorder, recurrent depression, substance dependence, or of any substance abuse in the last 6 months based on the SCID or (2) a current major depressive episode.

**Table 1:** **Mean ± standard deviation of demographic characteristics of participants**.

	Matching 1				Matching 2			
	Patients (*n* = 19)	Matched Control (*n* = 19)	*T/ χ^2^*	*P*	Sync Partners 1 (*n* = 19)	Sync Partners 2 (*n* = 19)	*T/χ^2^*	*P*
Age (years)	34.53 ± 12.59	35.89 ± 15.08	−0.30^*a*^	0.76	25.89 ± 4.11	24.79 ± 5.77	0.68^*a*^	0.50
Sex (Male/Female), *n*	12/7	6/13	3.80^*b*^	>0.05	5/14	10/9	2.75^*b*^	>0.05
Education (years)	13.58 ± 2.52	12.05 ± 2.30	1.95^*a*^	0.06	15.58 ± 1.89	15.89 ± 2.08	−0.49^*a*^	0.63
Premorbid IQ (f-NART)	110.95 ± 6.20	107.11 ± 8.01	1.65^*a*^	0.11	111.42 ± 7.92	107.89 ± 7.45	1.41^*a*^	0.17
LSAS Anxiety	40.21 ± 15.05	25.80 ± 15.21	2.94^*a*^	0.006	20.37 ± 9.15	19.79 ± 7.15	0.22^*a*^	0.83
LSAS Avoidance	32.37 ± 18.54	20.89 ± 11.67	2.28^*a*^	0.03	16.73 ± 9.25	12.26 ± 7.82	1.61^*a*^	0.12
LSAS Total	72.58 ± 31.56	46.69 ± 25.05	2.80^*a*^	0.008	37.11 ± 17.11	32.05 ± 13.77	1.00^*a*^	0.32

LSAS Liebowitz Social Anxiety Scale IQ Intellectual Quotient f-NART French version of the National Adult Reading Test

^a^Independent-samples *t*-test.

^b^Chi-square test.

To further investigate the link between social motor coordination impairments and SAD, the severity of SAD was evaluated for all participants using the self-report version of the Liebowitz Social Anxiety Scale (SR-LSAS; Fresco et al., 2001; Baker et al., 2002). The scale comprises 24 social situations that are each rated for level of fear (0 = none to 3 = severe) and avoidance (0 = none to 3 = usually). We used the LSAS Total score corresponding to the calculated sum of total fear and total avoidance scores (Stein et al., 1998; Kasper et al., 2005). All participants had normal or corrected-to-normal vision and provided written informed consent prior to the experiment, which was approved by the local Ethics Committee (CPP Sud Méditérannée III, Montpellier, France, AFSSAPS 2009-A00513-54 24, 07/22/2009) conforming to the Declaration of Helsinki.

### Apparatus

**Figure 1:**
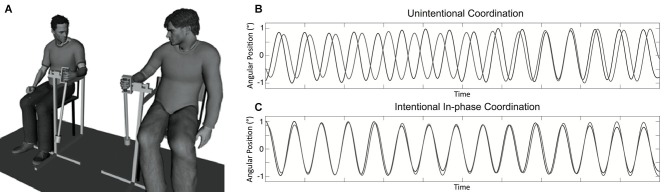
**Experimental setup and example of collected data**. Panel **(A)** displays the hand-held pendulum coordination task used in the study. Panel **(B)** represents a typical time series obtained for participants’ movement in the unintentional coordination task with spontaneous and intermittent synchronization over time toward in-phase and anti-phase patterns. Panel **(C)** represents a time series typically collected when participants had to synchronize their movements in an in-phase manner in the intentional coordination task.

Participants sat on chairs approximately 1 m from one another facing in the same direction and swung hand-held pendulums attached to a structure that allowed only movements from front to back (see Figure 1). The length of the two pendulums was 60 cm and a mass of 150 g was attached either to the bottom or middle of the length to constitute the pendulums P1 and P2, respectively. The preferred movement frequency of participants was 0.80 Hz for pendulum P1 and 0.95 Hz for pendulum P2 (averaged across participants in the first segments of unintentional coordination trials). Two potentiometers measured the angular displacements of pendulums during the trials at a sampling rate of 50 Hz.

### Design and procedure

Upon arrival, participants were informed that the experiment was investigating rhythmic movements with hand-held pendulums. Participants first performed practice trials individually, in which they were instructed to hold the pendulum firmly in their hand and to swing it at their own self-selected tempo, a tempo they found comfortable (“the tempo that you could swing all day if you had to”). Patients and *matched control participants* swung pendulums with their right hand and *synchronization partners 1* and *2* swung pendulums with their left hand. After the familiarization with the pendulums P1 and P2, participants were tested for social motor coordination.

We examined first the unintentional social motor coordination of participants. The participants performed three 90 s trials, one for each pendulum combination P2-P1, P1-P1 and P1-P2, corresponding respectively to the pendulums used by *synchronization partners 1* and patients for the SAD group and *synchronization partners 2* and *matched control* *participants* for the control group. Each trial consisted of three 30 s segments that were delimited by an auditory stimulus. Participants were instructed to oscillate their pendulum at the same self-selected tempo whether they were looking (Vision; second segment) or not looking (No vision; first and third segments) at the swinging of the other participant (Schmidt and O’Brien, 1997; Richardson et al., 2005; Oullier et al., 2008). During the first and third segments (i.e., no vision segments), participants had to focus on crosses located on the wall in the opposite direction of their partner. Participants were reminded just prior to each trial to swing the pendulum at their own self-selected tempo and to maintain this tempo throughout the trial.

The participants were then tested for intentional social motor coordination. They were informed that they would be required to coordinate their movements together in an in-phase or anti-phase manner for 60 s (Schmidt et al., 1998; Richardson et al., 2007). Both in-phase and anti-phase coordination were examined because previous research has shown that anti-phase coordination, being intrinsically less stable than in-phase, is more likely to be affected by pathologies (e.g., Varoqui et al., 2010). Participants performed three trials for each pattern of coordination, one for each of the three different pendulum combinations (i.e., P2-P1, P1-P1 and P1-P2). The order of these conditions was randomized.

### Coordination analysis

We discarded the first 5 s of each intentional trial as well as the first 5 s of each segment of the unintentional trials to avoid transient behavior. The time series records were low-pass filtered using a 10 Hz Butterworth filter. The continuous relative phase between the two angular positions of pendulums was computed using a Hilbert Transform (Pikovsky et al., 2003). For the intentional coordination conditions, from the computed relative phase, we calculated the standard deviation to assess the variability of the coordination performed as well as the phase shift from the intended pattern of coordination (Schmidt et al., 1998; Richardson et al., 2007). Positive phase shifts indicated that patients led the coordination whereas negative phase shifts indicated that patients followed the movements of the other participant. For the unintentional coordination conditions, we calculated the circular variance of the continuous relative phase which provides an index of synchronization from 0 (no synchronization) and 1 (perfect synchronization) (Batschelet, 1981; Tognoli et al., 2007; Oullier et al., 2008). The circular variance of the relative phase was used because the synchronization in unintentional situations is only intermittent and standard deviation measures are not appropriate to examine the variability of such nonstationary relative phase time series (Batschelet, 1981). We also computed the preferred movement frequency of participants as the inverse of the average time between the points of maximum angular extension as defined by the maxima of time series using the first segment (No vision) of the unintentional coordination.

### Statistical analysis

The circular variance values were standardized using a Fisher transformation. We then used a 2 × 3 × 3 repeated-measures analysis of variance (ANOVA) with the factors Group (Control, SAD), Pendulum Combination (P2-P1, P1-P1, P1-P2), and Segment (Segment 1 = “No vision”, Segment 2 = “Vision”, Segment 3 = “No vision”) to examine circular variance of the relative phase during unintentional coordination. We performed 2 × 3 × 2 repeated-measures analyses of variance (ANOVAs) with the factors Group (Control, SAD), Pendulum Combination (P2-P1 = “Follower”, P1-P1 = “Neutral”, P1-P2 = “Leader”), and Pattern (In-phase, Anti-phase) to examine the phase shift and standard deviation of relative phase during intentional coordination. *Post-hoc* Fisher LSD tests were used to determine the nature of the effect when necessary. One-sample *t*-tests were also used on the phase shift values to determine significant differences from zero (i.e., lagging or leading). Finally, in order to further explore the effect of the severity of SAD, we also performed correlational analyses to examine the relationship between the different motor coordination variables and the LSAS Total score obtained by SAD patients and *matched control participants* (*N* = 38). Those correlational analyses were performed on the whole sample because beyond the usual approach of considering SAD as a discrete category that is qualitatively distinct from normal social anxiety, we also considered SAD as part of the upper end of a continuum that differs only quantitatively (but not qualitatively) from normal social anxiety. Indeed, there is now growing evidence that social anxiety refers to a dimensional (lying along a continuum) rather than a categorical (representing a distinct entity) construct (Bögels et al., 2010; El-Gabalawy et al., 2010; Aderka et al., 2012b; Bunnell et al., 2013). The correlational analyses were performed using partial correlations with Group as between-subjects factor.

## Results

### Unintentional social motor coordination

The ANOVA performed on the circular variance values in the unintentional coordination task yielded significant main effects of Segment (*F*_(2,72)_ = 5.20, *p* = 0.008, $\eta_{p}^{2}=0.13$) and Pendulum Combination (*F*_(2,72)_ = 8.42, *p* < 0.001, $\eta_{p}^{2}=0.19$). In line with previous research, these results indicated that entrainment between participants’ movements increased when visually coupled (i.e., greater in Segment 2 = “Vision” than Segment 1 = “No vision” (*p* = 0.002) and Segment 3 = “No vision” (*p* = 0.08)) and when participants had preferred movement frequencies closer to each other (i.e., greater entrainment in P1-P1 than P1-P2 and P2-P1 (all *p-values* < 0.001)). However, this ANOVA did not reveal any effect of Group indicating that the SAD group synchronized as well as the control group when the coordination was unintentional. Moreover, no significant correlations were found between the LSAS Total score and movement synchronization during the visual interaction (Segment 2) (all *p-values* > 0.10), also suggesting that unintentional social motor coordination was not disrupted in SAD.

### Intentional social motor coordination

#### Phase shift from the intended coordination

**Figure 2:**
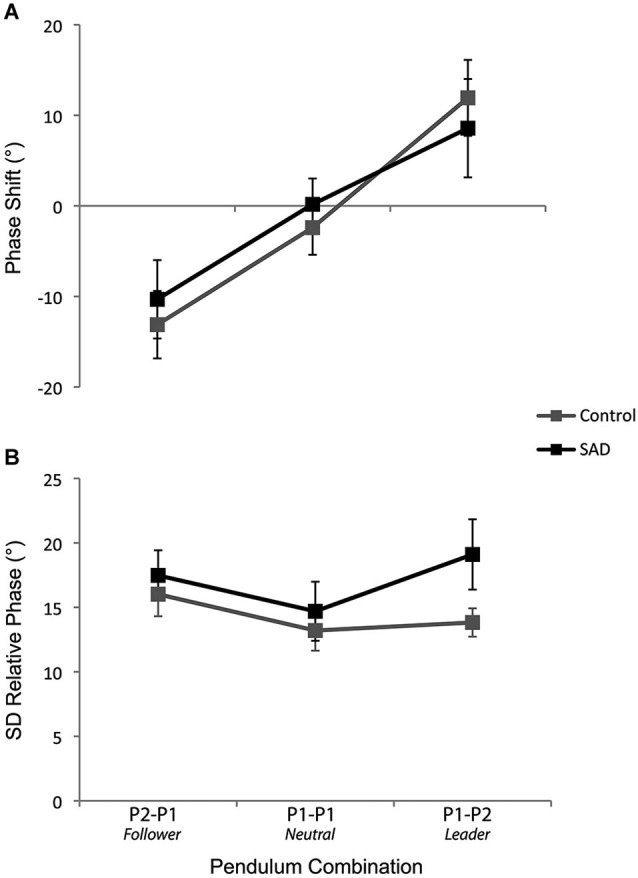
The phase shift from the intended coordination **(A)** and the standard deviation of relative phase **(B)** for the control and SAD groups as a function of the pendulum combination. The error bars represent the standard error of the mean.

The ANOVA performed on the phase shift values yielded a significant main effect of Pendulum Combination (*F*_(2,72)_ = 24.62, *p* < 0.001, $\eta_{p}^{2}=0.41$). One-sample *t*-tests indicated phase shifts were significantly negative in the P2-P1 (Follower) condition (*t*_(37)_ = −4.15, *p* < 0.001), not different from zero in the P1-P1 (Neutral) condition (*t*_(37)_ = −0.54, *p* = 0.59), and significantly positive in the P1-P2 (Leader) condition (*t*_(37)_ = 3.02, *p* = 0.005) (see Figure 2A). Corroborating previous studies, these results show that the participants who oscillated the pendulum with the fastest and slowest preferred movement frequencies led and followed the coordination, respectively. The ANOVA did not reveal any effect of Group (*F*_(1,36)_ = 0.2, *p* = 0.88, $\eta_{p}^{2}<0.001$). Correlational analyses did not reveal any significant relationships between the LSAS Total score and phase shifts values for any pendulum combinations (all *p-values* > 0.10), which confirms this last result and that social anxiety did not modulate the phase shifts from the intended coordination.

#### Standard deviation of the relative phase

The ANOVA performed on the standard deviation of the relative phase yielded a significant main effect of Pattern (*F*_(2,72)_ = 19.37, *p* < 0.001, $\eta_{p}^{2}=0.35$) and Pendulum Combination (*F*_(2,72)_ = 6.29, *p* = 0.003, $\eta_{p}^{2}=0.15$), effects that are in line with previous studies, namely, lower variability for in-phase compared to anti-phase coordination and lower variability when the pendulums were similar (i.e., P1-P1 lower than P2-P1 and P1-P2) (all *p-values* < 0.005). Interestingly, the analysis also demonstrated a significant interaction between Group and Pendulum Combination (*F*_(2,72)_ = 3.70, *p* = 0.03, $\eta_{p}^{2}=0.09$). Analysis of this interaction revealed more variable coordination for the SAD group compared to the control group in the condition in which the patient was leader (i.e., P1-P2 pendulum combination) (*p* = 0.049) (see Figure 2B).

**Figure 3:**
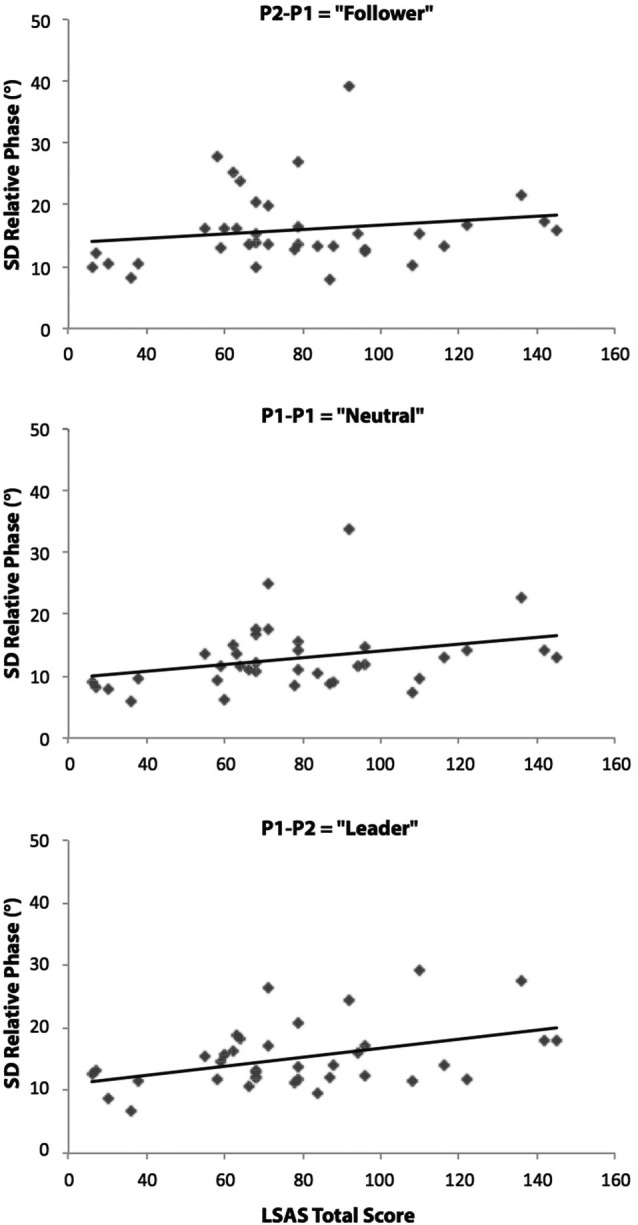
**Relationships between LSAS Total scores (SAD patients and *matched control participants*) and the relative phase standard deviations (average of the two phase patterns) for the P2-P1 (Follower), P1-P1 (Neutral) and P1-P2 (Leader) pendulum conditions**. Correlational analyses revealed significant positive linear relationships in P1-P1 and P1-P2 conditions (*p* < 0.05). The diagonal on the figures indicates the line of best fit.

The correlational analyses between the standard deviation of relative phase values (average of in-phase and anti-phase coordination) and LSAS Total scores for the three different pendulum combination conditions supported this last result. After removing an outliner in the SAD group (>3 standard deviations above the mean), the results yielded significant positive relationships between these two variables for the P1-P1 (Neutral) pendulum condition (*r*_(34)_ = 0.36; *p* = 0.03) and the P1-P2 (Leader) pendulum condition (*r*_(34)_ = 0.35; *p* = 0.03) (see Figure 3). No significant correlation was found in the P2-P1 (Follower) pendulum condition (*r*_(34)_ = 0.19; *p* = 0.27).

## Discussion

The goal of the current study was to investigate whether SAD affects the dynamics of social motor coordination. We compared unintended and intended rhythmic coordination of patients diagnosed with SAD (DSM-IV criteria) with the rhythmic coordination of control participants. We also examined the relationships between the severity of SAD as assessed by the LSAS and measures of social motor coordination. The results demonstrated that the coordination dynamics were disrupted in the SAD group when the coordination was intended and the SAD participant had to lead the coordination. Specifically, the rhythmic coordination was more variable for the SAD group than for the control group in this condition. We discuss this result in the next section as well as its implications for better understanding the origins of this mental disorder and improving its assessment and treatment.

It is important to observe that the disruption of social motor coordination occurred when the coordination was intentional but not when the coordination was unintentional. Such intended-unintended dissociation is in line with previous research that found a similar dissociation in social motor coordination in schizophrenia (Varlet et al., 2012b). Together, these studies suggest that social disorders might preferentially affect intentional rather than spontaneous unintended interpersonal coordination. More specifically for people with SAD, this result might be explained by the fact that intended coordination requires a social goal, and consequently, stronger social involvement. Important behavioral adaptations are needed to adequately maintain in-phase and anti-phase patterns of coordination and people with SAD might have been afraid to fail and to be judged (Lecrubier et al., 2000). Of particular interest with regard to this suggestion is that SAD group demonstrated the increased variability of social motor coordination only for some pendulum combinations. Stability became problematic in P1-P1 condition when the leader and follower positions were not mechanically constrained (significant correlation between the LSAS scores and the standard deviations of relative phase), and even more problematic when patients were constrained to lead the coordination (significant difference between the groups in the relative phase standard deviation ANOVA and significant correlation between LSAS scores and standard deviation of relative phase). The social coordination of the SAD group, however, was not more variable than the control group when the SAD participant was constrained to follow in the coordination, a condition in which patients might have been less afraid of being judged. This result supports past results that have found poorer leadership skills in patients with SAD and confirms the finding that anxiety can modulate motor performance (Weinberg and Hunt, 1976; Lecrubier et al., 2000; Bernstein et al., 2008; Hatfield et al., 2013).

Additional experiments remain necessary to better understand the coordination impairments of patients suffering from SAD. Manipulating explicitly the follower or leader position of patients (i.e., asking them to follow or lead the coordination, respectively) as well as measuring their anxiety in these conditions would be interesting to confirm the current results. It was also initially hypothesized that changes in the coordination of patients could originate from a motion perception deficit or irregular gaze behaviors. It would thus be interesting to examine the gaze behaviors of patients using eye-tracking systems (Roerdink et al., 2005) in leader and follower conditions. Finally, examining mediating effects of gender also appears necessary. In the current experiment, we tended to have more males in the SAD groups than in the control group. An ANOVA controlling for gender effects (i.e., gender of patients and *matched control participants* entered as covariate) and a potential confound did not result in any significant effects. However, the lack of a gender effect should be confirmed in future investigations with larger groups of participants. Such an effect is of particular interest in view of previous research that has shown differences in the social phobia of men and women, with greater fear of scrutiny for women than men (Turk et al., 1998). Moreover, it is also possible that such a potential gender effect depends on whether patients coordinate with a partner being the same or the opposite sex, which opens up interesting new research directions.

It is important to note that the behavioral pattern observed in SAD patients in the current study appears to be unique to this population. None of the previous studies that investigated social motor coordination in healthy subjects observed such modification of movement dynamics (Schmidt and Richardson, 2008; Schmidt et al., 2011). In contrast to the other factors that have affected rhythmic coordination such as visual attention or schizophrenia (Temprado and Laurent, 2004; Richardson et al., 2007; Schmidt et al., 2007; Varlet et al., 2012a), SAD only affected the variability of the coordination in specific leader-follower conditions and did this without affecting the phase shift from the intended coordination. The social motor coordination of patients with SAD seems distinguishable from those produced by reduced visual attention or schizophrenia. Therefore, these results suggest that such an impairment is an embodied signature of SAD and that interpersonal coordination measures could help the assessment of this mental disorder. These measures have the crucial advantage of providing a quantitative and objective assessment of nonverbal behavior disorders in patients and could thus complement structured interviews and surveys usually used (see Herbert et al., 2010 for a review) in which memory, reactivity, and social desirability biases of patients can be crucial issues (Kimberlin and Winterstein, 2008).

An additional confounding factor that should be investigated in future research is that the coordination exhibited in the current experiment also depended on the properties of the control partner, on his/her visual and attentional processes, his/her motivation and/or his/her reaction to their partner with social phobia. To evaluate such an issue, testing the coordination of patients with a virtual partner instead of a real partner would allow systematic control of the virual partner’s movement variance (Klinger et al., 2004, 2005; Wieser et al., 2010). This way the reaction of the other “person” can be controlled to isolate the effect of the person with the SAD on the rhythmic coordination that emerges during the interaction.

The disruption of the dynamics of social motor coordination demonstrated in the current study with SAD also raises important questions about the origins of these disorders. Do people with SAD have abnormal coordination dynamics because they have anxiety or do they have anxiety because they have abnormal coordination dynamics? Although the causal relation between bodily movement and mental states during social interactions is often unclear, there is growing evidence showing that bodily movement might be first in such a relation (Bernieri et al., 1994; Sebanz et al., 2006; Marsh et al., 2009; Wiltermuth and Heath, 2009). It has been demonstrated for instance that interpersonal bodily synchrony increases feelings of affiliation (Hove and Risen, 2009). People feel greater affiliation with someone else when moving in synchrony with him/her. In line with such a causal relationship between interpersonal coordination and participants’ mental states, we think that the movement coordination impairments of SAD patients could help create their anxiety disorder. Everyday people coordinate movements with others, for example, to move large and heavy objects, to walk through crowds, or to perform more complex group synchronization tasks as in musical or sport activities. In all these situations, having poorer leadership skills in joint-actions can decrease the success of the interactions, and might contribute in turn to the social anxiety of patients. This hypothesis warrants further explorations with synchronized behavioral and anxiety measures in order to examine such causal relationships.

Finally, if impaired social motor coordination of patients with SAD contributes to their mental illness and inevitably affects the success of their social exchanges, interventions aimed at improving the social motor coordination might lead to effective treatments. It is possible to develop rehabilitation protocols that can help patients with SAD to more successfully coordinate with others. Such rehabilitation may even be accelerated by using biofeedback and giving real-time information about their coordination with the others, which has been demonstrated in a variety of rehabilitation protocols (Kovacs et al., 2010; Kovacs and Shea, 2011; Varoqui et al., 2011; Peper et al., 2013). These protocols might help patients to adopt better natural coordination dynamics and better manage the leader position in motor coordination and interpersonal relations in general. Thus the present research suggests that social motor coordination rehabilitation protocols might efficaciously complement existing (cognitive, pharmacological) therapies for patients suffering from SAD by targeting improvement in the motor synchrony found in patients’ daily social interactions.

## Author contributions

Manuel Varlet, Ludovic Marin, Delphine Capdevielle, R. C. Schmidt, Robin N. Salesse, Benoît G. Bardy and Stéphane Raffard conceived and designed the experiment. Manuel Varlet, Delphine Capdevielle, Jonathan Del-Monte, Jean-Philippe Boulenger, and Stéphane Raffard performed the experiment. Manuel Varlet, Ludovic Marin, R. C. Schmidt, Robin N. Salesse, Benoît G. Bardy and Stéphane Raffard analyzed the data. All authors contributed to and have approved the final manuscript.

## Conflict of interest statement

The authors declare that the research was conducted in the absence of any commercial or financial relationships that could be construed as a potential conflict of interest.
